# The effectiveness of the combined problem-based learning (PBL) and case-based learning (CBL) teaching method in the clinical practical teaching of thyroid disease

**DOI:** 10.1186/s12909-020-02306-y

**Published:** 2020-10-22

**Authors:** Wanjun Zhao, Linye He, Wenyi Deng, Jingqiang Zhu, Anping Su, Yong Zhang

**Affiliations:** 1 Department of Thyroid Surgery, West China Hospital, Sichuan University, Chengdu, 610041 China; 2 West China School of Medicine, Sichuan University, Chengdu, 610041 China; 3 Key Laboratory of Transplant Engineering and Immunology, MOH; West China-Washington Mitochondria and Metabolism Research Center, West China Hospital, Sichuan University, 37 Guo Xue Xiang, Chengdu, 610041 NO China

**Keywords:** Problem-based learning, Case-based learning, Traditional lecture, Thyroid surgery, Clinical practical teaching

## Abstract

**Background:**

This study aimed to evaluate the effectiveness and efficiency of PBL–CBL combined teaching in thyroid surgery and make observations from the students’ perspectives, based on their satisfaction with the learning process.

**Methods:**

We prospectively enrolled 354 fourth-year students majoring in clinical medicine, along with 232 residents, from September 2014 to June 2019. These participants were randomly allocated into either the combined PBL–CBL teaching group or the traditional lecture-based classroom group to attend a course about thyroid nodules. Both pre- and post-class quizzes were conducted. An anonymous questionnaire was also administered to both groups to evaluate the students’ perceptions and experiences. We compared the two teaching methods among all the students as well as with the fourth-year students and residents in subgroups.

**Results:**

The traditional group’s pre-class quiz scores were significantly higher than the PBL–CBL group’s (as determined by a two-tailed t-test at a 95% confidence interval, *T* = 16.483, *P* < 0.001). After class, in the PBL–CBL group, the mean total quiz score and the basic knowledge and case analysis scores increased significantly (*P* < 0.001). The PBL–CBL group’s performance improvement was significantly higher than the traditional group’s (increasing from 52.76 to 70.51 vs. from 67.03 to 71.97). Furthermore, the scores for learning motivation, understanding, student–teacher interaction, the final examination, communication skills, clinical thinking skills, self-learning skills, teamwork skills, and knowledge absorption, as measured by the survey, were significantly higher in the PBL–CBL group than in the traditional group (*P* < 0.001). Meanwhile, the survey scores representing the amount of students’ free time the course consumed were significantly lower in the PBL–CBL group than in the traditional group (*P* < 0.001).

**Conclusions:**

PBL combined with CBL may be an effective method for improving medical students’ and residents’ performance and enhancing their clinical skills.

## Background

With medical and clinical knowledge and technology continuing to accumulate and advance, more and more international attention is focused on professional and normative medical education [1–3]. Medical colleges have long used a variety of approaches to develop effective teaching methods. Although a solid foundation of clinical knowledge and operational skills is important, the cultivation of medical students’ ability to perform medical case analysis as well as their on-the-spot response skills and capabilities have been elevated to new heights in recent years [4, 5].

Today, traditional lectures are still the most commonly adopted instructional method in medical and clinical teaching [6]. Lecturing is a popular way of teaching because it is both necessary and effective for transmitting core knowledge and concepts, especially to large audiences. However, despite the benefits of lecture-style teaching, research has produced evidence that shows that lectures are not effective for teaching important critical reasoning skills that are required in higher education, especially in professional courses, such as in the study of medicine. This is because the traditional lecture method [7, 8] is regarded as a teacher-centered educational approach whereby knowledge is transmitted by and from the teacher and passively received by the students.

Case-based learning (CBL) [9] is defined as a case-based education method that is grounded in the analysis of medical records with the aim of restoring the real clinical scene and prompting students to identify and develop new areas of learning. Problem-based learning (PBL) is defined as a student-centered pedagogy in which participants are allocated to groups of up to eight persons under non-directive tutors and given tasks or challenges that reflect situations that are relevant to the working environments they are anticipated to experience [10]. The PBL [11] teaching method advocates for students to solve problems through self-study, research, discussion, and cooperation within small groups, thereby cultivating students’ autonomous learning abilities and developing their comprehensive thinking capabilities; this represents a pedagogical shift from a teaching to a learning focus. Compared with traditional teaching methods, CBL is results-driven, with a focus on cultivating students’ rigorous logical reasoning. In real cases, teachers raise questions and students integrate their learned knowledge to analyze, deduce, and eventually solve problems. Thus, PBL is a problem-oriented, divergent-thinking education method that emphasizes students’ subjective initiative in learning; it is up to the students themselves to raise questions and work to solve them within small groups.

Many studies have found that CBL is effective in enhancing residents’ and medical students’ clinical practice, problem-solving, and analytical skills [12, 13]. Additionally, several recent systematic reviews have found that compared to traditional lectures, students in PBL programs consistently report higher levels of satisfaction and active engagement [14, 15]. However, taken alone, neither PBL nor CBL is without limitations [13, 16, 17]. CBL requires that teachers dedicate a lot of time to preparation in order to amass a sufficient number of cases to support clinical teaching. At the same time, CBL also demands that teachers create a set of questions for students to discuss, leading to a tendency for students to lack proactive involvement in and general enthusiasm for the learning experience. In contrast, PBL puts students in the central, leading role during the classroom process. This function requires them to spend a lot of time preparing problems and materials before class, which is extremely difficult for medical students, given their heavy curriculum tasks and commitments. In addition, PBL emphasizes students’ subjective initiative; however, a lack of guidance from teachers could lead to students missing the program focus, which could hinder general program quality. Therefore, we hypothesize that a teaching method that combines the virtues of PBL and CBL can better achieve the goal of promoting effective, high-quality student learning. To our knowledge, there is no literature that analyzes the outcome of a combined PBL–CBL method in medical education, especially with respect to teaching the topic of thyroid nodules in the Department of Thyroid Surgery, which is one of the most common diseases in endocrinal surgery.

To evaluate the effectiveness and acceptability of the combined PBL–CBL teaching method in thyroid surgery teaching, this paper reports on our implementation of the method among fourth-year students and residents in their thyroid surgery session over the past 5 years, drawing some comparisons between it and the traditional lecture teaching method. This study provides insights by examining students’ ways of reasoning in various areas, from basic knowledge to case analysis. Moreover, this study analyzes students’ perspectives regarding their self-perceived competence and their satisfaction with the PBL–CBL learning process.

## Methods

### Participants

This was a prospective, randomized, controlled study. We prospectively enrolled fourth-year students majoring in clinical medicine at the West China Medical College of Sichuan University and residents in the Department of Thyroid Surgery at the West China Hospital of Sichuan University from September 2014 to June 2019. They completed all the required thyroid disease courses that are provided at the West China Medical College and were taught by the same faculty. The participants were randomly sorted into either the “combination group,” featuring a PBL–CBL combined teaching program, or the “traditional group,” featuring a lecture-based teaching program. The students were kept unaware of their group assignments prior to their internships. A simple randomization was adopted for this study. Since the courses were arranged at different times, students and residents who took class at the same time were organized in ascending order by their identification numbers. All students and residents were renumbered as 1 to N. If the assigned number was odd, he/she entered the PBL–CBL group, whereas if the number was even, he/she entered the traditional group. Each group was supervised by teaching staff consisting of one instructor and three assistants who held full-time professional positions within the Department of Thyroid Surgery. Informed consent was obtained from all participants. The study was approved by the Institutional Review Board and Ethics Committee of the West China Hospital of Sichuan University.

### Study design

We chose thyroid nodules as the topic for applying the combined PBL–CBL approach in this study because the diagnosis and treatment of thyroid nodules is one of the key courses that students must master in the Department of Thyroid Surgery.

The PBL–CBL group’s program was arranged as follows. Before class, the instructor prepared lecture videos and supplementary materials for the course. The students were given general diagnosis and treatment guidelines (Chinese and English versions), five reference papers related to the course’s topics, and roughly 30 min of video materials on operational procedures. Each student was required to review these materials in his/her own free time outside of class. Before the classroom activities began, the students were asked to complete a pre-class quiz consisting of 32 multiple-choice questions about thyroid nodules. The class session was prefaced with the instructor providing a brief introduction of the topic and the class agenda. Next, as a first step in the classroom activities, a patient case with slides was presented. Second, the students carried on small-group discussions under the instructor’s guidance. During these discussions, the participants were encouraged to raise relevant questions and seek answers on the Internet and in the library database. Third, a student representative from each group gave a presentation to review the main points from the lesson, share their group’s answers to the questions posed, and ask about any unsolved questions. Finally, the instructor summarized the class and went over the tough questions that were raised during discussion. At the end of these classroom activities, the students were asked to complete a post-class quiz consisting of the same questions about thyroid nodules that appeared in the pre-class quiz. They were also asked to complete a survey consisting of 10 questions about their perceptions and experiences in the combined PBL–CBL classroom.

The traditional lecture group program was as follows. Before the lecture, the students were instructed to simply preview the course, instead of watching videos or reading materials in any extensive way. They also took the same pre-class quiz (consisting of the same 32 multiple-choice questions) that was administered to the PBL–CBL group. These students were taught the equivalent content via the traditional teaching method; that is, the instructor provided a thorough explanation of the theoretical knowledge within the official framework, instead of dividing the class into small groups to discuss the cases. In other words, instructor teaching was the predominant approach. After class, the students took the same post-class quiz (containing the same questions) as the one described above for the PBL–CBL group; they also completed the same survey as the PBL–CBL group.

**Fig. 1:**
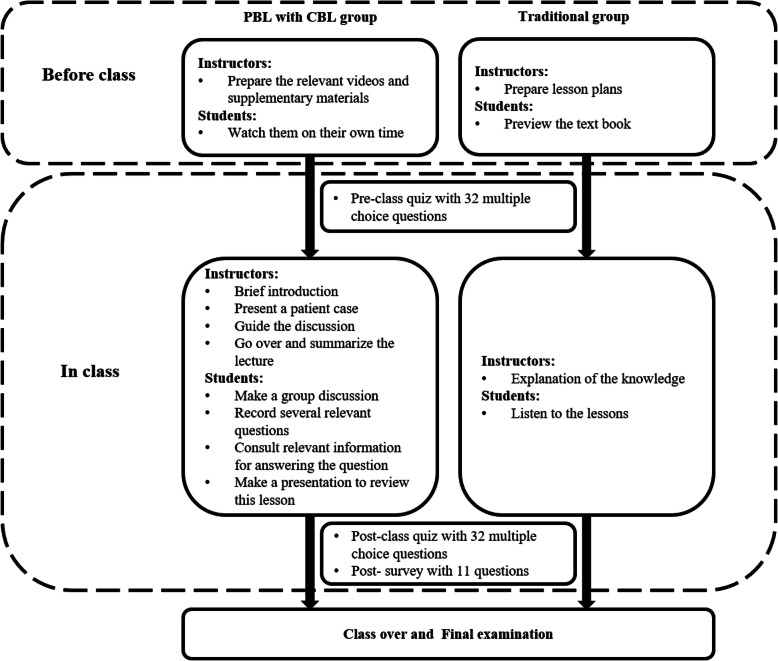
An overview of the study design

All the students were given consent forms and informed that their participation in the quizzes and the survey was voluntary. Since identification numbers were used in the quizzes and the survey instead of real names, the quiz and survey results had no (positive or negative) effect on students’ course grades or performance. The students completed the quizzes and the survey independently of their peers and the teaching staff. A graphical overview of the study design is shown in Fig. 1.

### Data evaluation and statistical analysis

Both the pre- and post-class quizzes were conducted to evaluate what the students gained from the thyroid nodule course. The quizzes were composed of basic theoretical questions (worth 50 points) and clinical case analyses (worth 50 points). All the questions were based on Bloom’s Taxonomy, [18, 19] which categorizes cognitive activities into six hierarchical levels, namely, memory, understanding, application, analytical skills, assessment, and creativity. The “remembering” and “understanding” categories were combined into a single category called “basic theoretical knowledge.” Items in any of the other categories were considered “clinical case analyses.”

After class, students from both groups were required to complete the same anonymous questionnaire to evaluate their perceptions and experiences. The post-class survey consisted of 10 questions, including [20] questions about motivation, understanding, student–teacher interaction, how much free time the course consumed, the final examination, communication skills, clinical thinking skills, self-learning skills, teamwork skills, and knowledge absorption. The evaluation criteria were based on a previous study. Based on the degree of improvement, the scores were divided into five grades, from 1 (poor) to 5 (excellent). In contrast to the other scored areas, for free time consumed, 1 represents the lowest time consumption, while 5 indicates the highest. The reliability of the questionnaire was evaluated. The Cronbach’s alpha coefficient was 0.872.

The amount of time that students spent preparing before class was measured for each group. In the PBL–CBL group, students’ preparation time was recorded as having been spent watching course-related lecture videos and reading materials as well as searching for supplemental materials on the Internet. Meanwhile, for the traditional group, students’ preparation time was recorded as having been spent previewing the textbook.

We compared the effectiveness of PBL–CBL versus traditional teaching methods across all the fourth-year students and residents. In addition, in order to avoid bias resulting from different types of students, we also compared the two teaching methods with fourth-year students and residents in subgroups.

We compiled the total scores and then compared the results generated by each of the two groups using an independent sample T-test. We also compared the data that were generated by the groups before and after class using a paired sample T-test. The chi-square test was used to compare the rates. All statistical analyses were performed using SPSS version 20.0 (Chicago, USA). Alpha was set at 0.05, and *P*-values of less than 0.05 were considered significant.

## Results

### Basic characteristics and information

A total of 354 fourth-year students and 232 residents were enrolled from September 2014 to June 2019. In total, 97.2% (344/354) of the fourth-year students and 97.0% (225/232) of the residents completed the pre- and post-class quizzes and survey, including 276 participants (167 fourth-year students and 109 residents) who were assigned to the PBL–CBL group and 293 (177 fourth-year students and 116 residents) who were assigned to the traditional group. Seventeen students in the PBL–CBL group did not complete the program. Of these, 11 initially signed the informed consent to participate in this study but withdrew before class. Therefore, although these students took part in the traditional lectures, they were excluded from the study. The other six students were also excluded because the missing values in their quizzes and surveys exceeded 50%. The mean age of the students was 21.43 ± 1.389. Among them, there were 289 female students, accounting for 50.8%. Table 1 compares the basic student characteristics in the PBL–CBL group and the traditional group. There were no significant differences between the two groups in terms of gender, age, or grades (*P* > 0.05). Additionally, for the fourth-year students and residents, the differences in terms of the demographic characteristics of those students who were in the PBL–CBL group versus the traditional group showed no statistical significance (shown in Table S1). The average time spent on pre-class preparation in the PBL–CBL and traditional groups was 107.23 ± 14.512 and 95.60 ± 15.631, respectively. Evidently then, compared to the traditional group, the PBL–CBL group members spent significantly more time preparing before class than the students in the traditional group (*P* < 0.001).

**Table 1:** The basic characteristics of all the participants

Item	PBL–CBL group (***N*** = 276)	Traditional group (***N*** = 293)	Statistics	***P*** value
**Grade**			χ2 = 0.001	0.981
Fourth-year students	167	177		
Residents	109	116		
**Gender**			χ2 = 0.525	0.469
Male	145	144		
Female	131	149		
**Age**	21.49 ± 1.443	21.39 ± 1.354	T = 0.850	0.396

### The comparison of quiz scores between the PBL–CBL and traditional groups

**Fig. 2:**
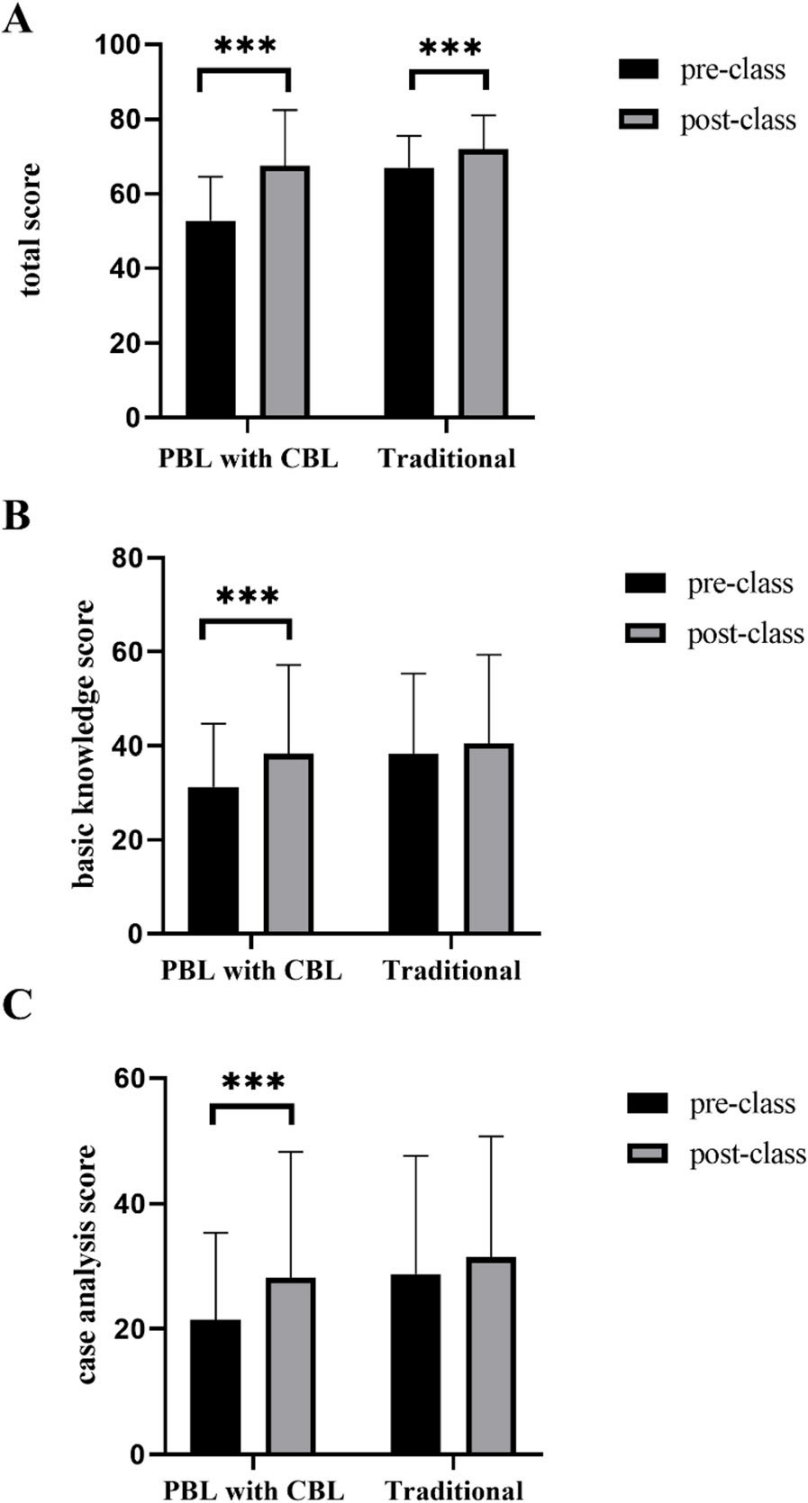
The comparison of pre- and post-class quiz scores in the PBL–CBL and traditional groups. **a**. The comparison of the total pre- and post-class quiz scores (**b)**. The comparison of the basic knowledge pre- and post-class quiz scores (**c**). The comparison of the case analysis pre- and post-class quiz scores. *** means *p* < 0.001

We compared the PBL–CBL and traditional groups’ pre- and post-class quiz scores (shown in Table 2). In the PBL–CBL group, the mean pre-class total quiz score and the basic knowledge and case analysis scores were 52.76 ± 11.778, 31.28 ± 13.435, and 21.49 ± 13.899, respectively. Meanwhile, for the traditional group, they were 67.03 ± 8.506, 38.25 ± 17.104, and 28.78 ± 18.779, respectively. It is notable that the traditional group’s pre-class quiz scores were significantly higher than the PBL–CBL group’s (*P* < 0.001). After class, the mean total quiz score and the basic knowledge and case analysis scores for the PBL–CBL group increased significantly, from 52.76 to 70.51, 31.28 to 38.33, and 21.48 to 29.18, respectively (*P* < 0.001) (Fig. 2). Similarly, in the traditional group, the mean total quiz score increased significantly from 67.03 to 71.97 (*P* < 0.01); however, the basic knowledge and case analysis scores improved from 38.25 to 40.49 and from 28.78 to 31.47, respectively, which was not statistically significant (*P* = 0.150 and *P* = 0.086, respectively). Furthermore, there was no significant difference in terms of the post-class quiz scores between the PBL–CBL and the traditional groups. We also performed a subgroup analysis of the fourth-year students and residents, which yielded the same results as the full student group (shown in Fig. 2, Table S2, and Table S3).

**Table 2:** The comparison of the pre- and post-class test scores of the PBL–CBL group vs. the traditional group (for all participants)

Item	PBL–CBL group (***N*** = 276)	Traditional group (***N*** = 293)	***T***	***P*** value
**Total pre-class score**	52.76 ± 11.778	67.03 ± 8.506	16.483	<0.001
**Pre-class basic knowledge score**	31.28 ± 13.435	38.25 ± 17.104	5.422	<0.001
**Pre-class case analysis score**	21.48 ± 13.899	28.78 ± 18.779	5.291	<0.001
**Total post-class score**	70.51 ± 14.561	71.97 ± 9.096	1.423	0.155
**Post-class basic knowledge score**	38.33 ± 18.808	40.49 ± 18.869	1.370	0.171
**Post-class case analysis score**	29.18 ± 20.043	31.47 ± 19.190	1.388	0.165

### The comparison of survey scores between the PBL–CBL and traditional groups

We compared the post-class survey scores pertaining to students’ perspectives and self-perceived competence in the PBL-CBL and traditional groups and found that the scores for learning motivation, understanding, student–teacher interaction, the final examination, communication skills, clinical thinking skills, self-learning skills, teamwork skills, and knowledge absorption were significantly higher in the PBL–CBL group than in the traditional group (*P* < 0.001) (Table 3). Meanwhile, the survey scores representing how much of the students’ free time the coursework consumed (combining the time spent doing both pre- and post-class work) were significantly lower for the PBL–CBL group than for the traditional group (*P* < 0.001) (Table 3). We also performed a subgroup analysis of fourth-year students and residents (Table S4 and S5), which yielded results that were consistent with those for all the students.

**Table 3:** The comparison of perspectives and self-perceived competence in the PBL–CBL group vs. the traditional group (for all participants)

Item	PBL–CBL group (***N*** = 276)	Traditional group (***N*** = 293)	***T***	95% confidence interval	***P*** value
**Learning motivation (point)**	4.03 ± 0.813	3.06 ± 0.844	13.977	0.835,1.107	<0.001
**Understanding (point)**	4.05 ± 0.812	2.94 ± 0.836	16.048	0.978,1.244	<0.001
**Student–teacher interaction (point)**	3.98 ± 0.786	3.02 ± 0.794	14.460	0.828,1.088	<0.001
**Free time consumed (point)**	2.07 ± 0.838	2.94 ± 0.825	14.481	0.874,1.148	<0.001
**Final examination (point)**	4.01 ± 0.788	3.05 ± 0.801	14.352	0.825,1.087	<0.001
**Communication skills (point)**	4.00 ± 0.825	2.49 ± 0.501	26.305	1.402,1.629	<0.001
**Clinical thinking skills (point)**	4.01 ± 0.822	2.48 ± 0.501	26.671	1.422,1.646	<0.001
**Self-learning skills (point)**	3.98 ± 0.824	2.58 ± 0.495	24.465	1.292,1.646	<0.001
**Teamwork skills (point)**	4.02 ± 0.802	2.58 ± 0.494	25.559	1.327,1.548	<0.001
**Knowledge absorption (point)**	4.02 ± 0.807	2.46 ± 0.499	27.560	1.449,1.672	<0.001

### The comparison of the learning effect factors between the satisfied and unsatisfied groups

In order to further evaluate the factors that influenced the participants’ learning experiences, we classified the post-class quiz scores such that those that were greater than or equal to 80 points were defined as satisfactory. For the PBL–CBL group, the scores for understanding, communication skills, clinical thinking skills, self-learning skills, teamwork skills, and knowledge absorption in the satisfied group (≥ 80 scores) were higher than those in the unsatisfied group; however, there was no statistically significant difference between the two groups (Table 4). Additionally, in the traditional group, the scores for the same areas as mentioned above for the satisfied group were higher than those in the unsatisfied group. There were also no statistically significant (shown in Table 5).

**Table 4:** The comparison of perspectives and self-perceived competence between the satisfied and unsatisfied groups (PBL–CBL group)

Item	≥80 scores (***n*** = 65)	<80 scores (***n*** = 211)	***T***	***P*** value
**Understanding (point)**	4.17 ± 0.821	4.01 ± 0.808	1.336	0.184
**Communication skills (point)**	4.06 ± 0.768	3.99 ± 0.842	0.679	0.498
**Clinical thinking skills (point)**	4.06 ± 0.827	4.00 ± 0.822	0.525	0.599
**Self-learning skills (point)**	4.05 ± 0.799	3.96 ± 0.833	0.734	0.464
**Teamwork skills (point)**	4.16 ± 0.756	4.05 ± 0.815	1.137	0.258
**Knowledge absorption (point)**	4.05 ± 0.818	4.01 ± 0.805	0.317	0.752

**Table 5:** The comparison of perspectives and self-perceived competence between the satisfied and unsatisfied groups (Traditional group)

Item	≥80 scores (***n*** = 65)	<80 scores (***n*** = 211)	***T***	***P*** value
**Understanding (point)**	3.05 ± 0.873	2.97 ± 0.823	1.090	0.278
**Communication skills (point)**	2.50 ± 0.503	2.49 ± 0.501	0.177	0.860
**Clinical thinking skills (point)**	2.54 ± 0.502	2.46 ± 0.500	1.042	0.300
**Self-learning skills (point)**	2.67 ± 0.501	2.59 ± 0.493	1.077	0.284
**Teamwork skills (point)**	2.65 ± 0.501	2.59 ± 0.493	0.599	0.550
**Knowledge absorption (point)**	2.46 ± 0.502	2.45 ± 0.499	0.144	0.886

## Discussion

The traditional lecture teaching method is indeed the most economical and efficient way to deliver a theoretical lecture [21, 22]; however, it is not suitable for high-grade medical students, who need to cultivate superior communication and clinical thinking skills. With the advent of the Internet, information is growing explosively, and since personal computers and mobile devices have made e-learning a part of tertiary medical education, helping medical students actively obtain effective information within limited timeframes and allowing them to actively think and ask questions, while guiding them in the acquisition of new information are all extremely important teaching components [17, 23]. In this endeavor, PBL and CBL, which are markedly different from traditional teaching methods, aim to establish real medical scenes and encourage students to take subjective initiative toward shifting from a “what I have been taught” paradigm to “what I want to learn.” [24] Most previous studies have focused on either PBL or CBL separately [25–31]. Compared with traditional lecture teaching methods, some studies have even demonstrated the advantages of either PBL or CBL. For instance, PBL has established the small-group learning mode, which features more thorough teacher–student communication and thus can achieve personalized teaching goals [32]. Meanwhile, through the preparation of clinical case materials, CBL emphasizes teacher guidance to help students form more effective comprehensive clinical thinking habits [33]. In light of these separate merits, we combined the PBL and CBL teaching methods in this study, so that the two could complement and reinforce each other.

We investigated the combined PBL–CBL teaching method’s effectiveness and acceptability in a clinical course on thyroid nodules through a comparison with the traditional lecture teaching method. To our knowledge, combined PBL–CBL teaching had not been previously implemented in thyroid surgery classes with a large number of participants. In our study, the total pre-class quiz score for the traditional group was significantly higher than the PBL–CBL group’s, indicating the benefit of pre-class previews. Before class, the students in the PBL–CBL group browsed through their course materials, which did not include detailed information about cases for analysis and discussion topics. In contrast, the students in the traditional lecture group memorized basic knowledge from their textbook. We therefore conclude that the difference in pretest scores between the two groups was not due to pre-class material quality but can be attributed instead to the two teaching methods’ different characteristics. Specifically, given that the pre-class PBL–CBL course materials are more abundant and more closely approximate clinical work, the PBL–CBL students’ focus on knowledge points was relatively weak, whereas the students in the traditional lecture group were more likely to have found and memorized knowledge points in their textbooks.

However, by comparing the total pre- and post-class quiz scores, we found that the PBL–CBL group’s performance improvement was significantly higher than the traditional group’s (from 52.76 to 67.51 vs. from 67.03 to 71.97), thus indicating the effectiveness of the combined PBL–CBL teaching model. Of course, the inevitability of a possible ceiling effect is notable. That is, once a certain (high) score is achieved prior to a student’s exposure to educational content, it is more difficult to improve this score than if a low score is initially obtained. Additionally, although there was no significant statistical difference between the two groups in terms of the post-class quiz scores, the PBL–CBL combined teaching method occupied less of the students’ free time, which evidences its efficiency for application to medical education.

In our study, all the quiz questions were based on Bloom’s Taxonomy, [34] which is widely used in education research to stratify learning activities into different cognitive levels, ranging from basic recall to higher educational objectives such as memory, understanding, application, analytical skills, assessment, and creativity [34]. The textbook was the main source of basic knowledge, requiring the students to read and memorize. Meanwhile, for case analysis, students must analyze the cases in the context of a relatively realistic medical scene in which they utilize the knowledge they have acquired to attempt to solve real-world medical and clinical problems. We further analyzed the PBL–CBL group’s scores, and the results showed that their clinical case analysis scores improved more significantly than their basic knowledge scores, meaning that the combined PBL–CBL teaching model is more conducive for cultivating creative thinking and is also more consistent with the general goals of medical teaching. According to the analysis of students’ perspectives and self-perceived competence as measured by the survey in the two groups, we confirmed that the students in the PBL–CBL group tended to take a more well-balanced approach to learning and practice, thus becoming more proactive learners. The combined PBL–CBL teaching model’s positive impact on students in the curriculum areas of understanding, communication skills, clinical thinking skills, self-learning skills, teamwork skills, and knowledge absorption was also well received by the participants themselves.

Previous studies have shown that there have been attempts to implement either the PBL or the CBL teaching model in the delivery of various college and university majors, [9, 11, 13, 21] but few have paid attention to combined PBL–CBL teaching in clinical medicine. Ginzburg et al. applied the combined PBL–CBL teaching method to medical students’ discussions about cost-related healthcare topics, showing that PBL combined with CBL is an effective method for engaging in conversations related to public health [35]. Another study implemented the combined PBL–CBL teaching method in six courses to improve students’ leadership skills without occupying curricular training time [36]. Moreover, Hu et al. combined the flipped classroom with PBL in a hyperthyroidism course, which achieved improved performance, albeit at the cost of a heavier workload for students [37]. This study is generally consistent with the above-mentioned studies, indicating that the combined method improves student performance. Furthermore, as far as the amount of time consumed, we agree with Ginzburg, [36] but we differ from Hu et al. [37] Furthermore, we differ from all of the above studies in that we conducted a more in-depth study of the different skills that students can learn through the combined PBL–CBL teaching method, and we performed a subgroup analysis of the different types of students, including fourth-year students and residents. In sum, our study confirmed the effectiveness of the combined PBL–CBL teaching method with respect to improving students’ general understanding of the professional field, student–teacher interaction, communication skills, clinical thinking skills, self-learning skills, teamwork skills, and knowledge absorption.

However, our study had several limitations. First, we analyzed results from only one clinical department within our institution; these results may have been different beyond our institution. Second, since there was no blind method in our study, some analysis bias is unavoidable. Given that the nature of the curriculum prevents facilitators from observing students blindly, we acknowledge that their assessments of students’ leadership traits may have been influenced by subjective factors, including improved perception and interpersonal bonds that formed over time. Therefore, satisfaction survey content may be biased toward PBL–CBL in the areas of self-learning and teamwork skills. Third, our study was based on one thyroid nodules course. Long-term observation and practice would make the study more robust, while producing more grounded assessments. In the future, we will conduct an experiment with multiple central randomized trials, a large sample size, and long-term follow-up.

## Conclusion

In conclusion, the combined PBL–CBL teaching method may be effective for improving medical students’ and residents’ performance and enhancing their clinical skills and capabilities when learning about thyroid nodules in the Department of Thyroid Surgery. The method resulted in better pre-class preparation and the immediate provision of feedback. Additionally, the combined PBL–CBL teaching method effectively enhanced students’ understanding, student–teacher interaction, communication skills, clinical thinking skills, self-learning skills, teamwork skills, and knowledge absorption.

## Supplementary information

**Additional file 1: Table S1.** The basic characteristics of the fourth-year students and residents.

**Additional file 2: Table S2.** The comparison of the pre- and post-class test scores of the PBL–CBL and the traditional groups (fourth-year students).

**Additional file 3: Table S3.** The comparison of the pre- and post-class test scores of the PBL–CBL and traditional groups (residents).

**Additional file 4: Table S4.** The comparison of perspectives and self-perceived competence between the PBL–CBL and traditional groups (fourth-year students).

**Additional file 5: Table S5.** The comparison of perspectives and self-perceived competence between the PBL–CBL and traditional groups (residents).

**Additional file 6.**

## Data Availability

The original data were deposited into the Mendeley Data dataset (https://data.mendeley.com/datasets) with **DOI:** 10.17632/7484xmwk46.1. The questionnaire used in this study refers to the articles published in Ref. 20 [20].

## Abbreviations

PBL: Problem-based learning
CBL: Case-based learning
