# Supplementary material for: The effectiveness of the combined problem-based learning (PBL) and case-based learning (CBL) teaching method in the clinical practical teaching of thyroid disease
Source: BMC Med Educ. 2020 Oct 22;20:381. doi: 10.1186/s12909-020-02306-y (PMC7583209; doi:10.1186/s12909-020-02306-y)
Supplement: Supplementary file 1 — Additional file 1: Table S1. The basic characteristics of the fourth-year students and residents. [file 12909_2020_2306_MOESM1_ESM.docx]

| **Table S1.** The basic characteristics of the fourth-year students and residents | | | | | | | | |
| --- | --- | --- | --- | --- | --- | --- | --- | --- |
| **Item** | **Fourth-year students (*N* = 344)** | | | | **Residents (*N* = 225)** | | | |
|  | **PBL–CBL group (*N* = 167)** | **Traditional group (*N* = 177)** | **Statistics** | ***P* value** | **PBL–CBL group (*N* = 109)** | **Traditional group (*N* = 116)** | **Statistics** | ***P* value** |
| **Gender** |  |  | χ2=0.595 | 0.441 |  |  | χ2=0.004 | 0.948 |
| Male | 90 | 87 |  |  | 55 | 57 |  |  |
| Female | 77 | 90 |  |  | 54 | 59 |  |  |
| **Age** | 20.54±0.782 | 20.49±0.986 | T=0.623 | 0.534 | 22.94±0.926 | 22.75±0.986 | T=1.458 | 0.146 |
