# Supplementary material for: The effectiveness of the combined problem-based learning (PBL) and case-based learning (CBL) teaching method in the clinical practical teaching of thyroid disease
Source: BMC Med Educ. 2020 Oct 22;20:381. doi: 10.1186/s12909-020-02306-y (PMC7583209; doi:10.1186/s12909-020-02306-y)
Supplement: Supplementary file 2 — Additional file 2: Table S2. The comparison of the pre- and post-class test scores of the PBL–CBL and the traditional groups (fourth-year students). [file 12909_2020_2306_MOESM2_ESM.docx]

| **Table S2.** The comparison of the pre- and post-class test scores of the PBL–CBL and the traditional groups (fourth-year students) | | | | | | |  |
| --- | --- | --- | --- | --- | --- | --- | --- |
| **Item** | **PBL–CBL group (*N* = 167)** | **Traditional group (*N* = 177)** | | ***T*** | | ***P* value** | |
| **Total pre-class score** | 49.46±11.664 | 65.04±9.154 | 13.727 | | ＜0.001 | |  |
| **Pre-class basic knowledge score** | 29.90±13.408 | 38.76±17.417 | 5.300 | | ＜0.001 | |  |
| **Pre-class case analysis score** | 19.56±13.225 | 26.28±19.180 | 3.804 | | ＜0.001 | |  |
| **Total post-class score** | 69.67±14.725 | 69.92±9.702 | 0.978 | | 0.782 | |  |
| **Post-class basic knowledge score** | 37.53±18.464 | 39.62±19.044 | 1.036 | | 0.301 | |  |
| **Post-class case analysis score** | 29.14±18.562 | 30.29±18.981 | 1.316 | | 0.134 | |  |
