# Supplementary material for: The effectiveness of the combined problem-based learning (PBL) and case-based learning (CBL) teaching method in the clinical practical teaching of thyroid disease
Source: BMC Med Educ. 2020 Oct 22;20:381. doi: 10.1186/s12909-020-02306-y (PMC7583209; doi:10.1186/s12909-020-02306-y)
Supplement: Supplementary file 3 — Additional file 3: Table S3. The comparison of the pre- and post-class test scores of the PBL–CBL and traditional groups (residents). [file 12909_2020_2306_MOESM3_ESM.docx]

| **Table S3.** The comparison of the pre- and post-class test scores of the PBL–CBL and traditional groups (residents) | | | | |
| --- | --- | --- | --- | --- |
| **Item** | **PBL–CBL group (*N* = 109)** | **Traditional group (*N* = 116)** | ***T*** | ***P* value** |
| **Total pre-class score** | 57.81±10.078 | 79.06±6.335 | 10.840 | ＜0.001 |
| **Pre-class basic knowledge score** | 33.38±13.263 | 37.47±16.660 | 2.043 | 0.42 |
| **Pre-class case analysis score** | 24.43±14.442 | 32.59±17.553 | 3.819 | ＜0.001 |
| **Post-class total score** | 73.40±13.157 | 75.09±7.049 | 1.191 | 0.227 |
| **Post-class basic knowledge score** | 39.56±19.344 | 41.83±18.603 | 0.896 | 0.371 |
| **Post-class case analysis score** | 33.84±21.379 | 33.27±19.450 | 0.212 | 0.832 |
