# Supplementary material for: The effectiveness of the combined problem-based learning (PBL) and case-based learning (CBL) teaching method in the clinical practical teaching of thyroid disease
Source: BMC Med Educ. 2020 Oct 22;20:381. doi: 10.1186/s12909-020-02306-y (PMC7583209; doi:10.1186/s12909-020-02306-y)
Supplement: Supplementary file 4 — Additional file 4: Table S4. The comparison of perspectives and self-perceived competence between the PBL–CBL and traditional groups (fourth-year students). [file 12909_2020_2306_MOESM4_ESM.docx]

| **Table S4.** The comparison of perspectives and self-perceived competence between the PBL–CBL and traditional groups (fourth-year students) | | | | |  |
| --- | --- | --- | --- | --- | --- |
| **Item** | **PBL–CBL group (*N* = 167)** | **Traditional group (*N* = 177)** | ***T*** | ***P* value** | |
| **Learning motivation (point)** | 4.09±0.805 | 3.03±0.829 | 12.049 | ＜0.001 | |
| **Understanding (point)** | 4.04±0.798 | 2.98±0.849 | 11.853 | ＜0.001 | |
| **Student–teacher interaction (point)** | 2.05±0.795 | 2.97±0.790 | 11.745 | ＜0.001 | |
| **Free time consumed (point)** | 3.92±0.821 | 2.97±0.829 | 10.680 | ＜0.001 | |
| **Final examination (point)** | 4.00±0.760 | 3.08±0.790 | 10.951 | ＜0.001 | |
| **Communication skills (point)** | 4.01±0.821 | 2.49±0.501 | 20.578 | ＜0.001 | |
| **Clinical thinking skills (point)** | 4.04±0.824 | 2.51±0.501 | 20.629 | ＜0.001 | |
| **Self-learning skills (point)** | 3.93±0.840 | 2.55±0.499 | 18.319 | ＜0.001 | |
| **Teamwork skills (point)** | 3.90±0.816 | 2.58±0.495 | 18.049 | ＜0.001 | |
| **Knowledge absorption (point)** | 4.08±0.810 | 2.43±0.496 | 22.688 | ＜0.001 | |
