# Supplementary material for: The effectiveness of the combined problem-based learning (PBL) and case-based learning (CBL) teaching method in the clinical practical teaching of thyroid disease
Source: BMC Med Educ. 2020 Oct 22;20:381. doi: 10.1186/s12909-020-02306-y (PMC7583209; doi:10.1186/s12909-020-02306-y)
Supplement: Supplementary file 5 — Additional file 5: Table S5. The comparison of perspectives and self-perceived competence between the PBL–CBL and traditional groups (residents). [file 12909_2020_2306_MOESM5_ESM.docx]

| **Table S5.** The comparison of perspectives and self-perceived competence between the PBL–CBL and traditional groups (residents) | | | | |
| --- | --- | --- | --- | --- |
| **Item** | **PBL–CBL group (*N* = 109)** | **Traditional group (*N* = 116)** | ***T*** | ***P* value** |
| **Learning motivation (point)** | 3.94±0.820 | 3.10±0.869 | 7.395 | ＜0.001 |
| **Understanding (point)** | 4.07±0.836 | 2.88±0.815 | 10.843 | ＜0.001 |
| **Student–teacher interaction (point)** | 3.99±0.776 | 3.10±0.795 | 8.470 | ＜0.001 |
| **Free time consumed (point)** | 2.16±0.866 | 2.89±0.821 | 9.789 | ＜0.001 |
| **Final examination (point)** | 4.03±0.833 | 3.01±0.818 | 9.249 | ＜0.001 |
| **Communication skills (point)** | 3.99±0.833 | 2.48±0.502 | 16.317 | ＜0.001 |
| **Clinical thinking skills (point)** | 3.97±0.822 | 2.43±0.497 | 16.892 | ＜0.001 |
| **Self-learning skills (point)** | 4.06±0.797 | 2.61±0.489 | 16.351 | ＜0.001 |
| **Teamwork skills (point)** | 4.19±0.751 | 2.58±0.496 | 18.904 | ＜0.001 |
| **Knowledge absorption (point)** | 3.92±0.795 | 2.50±0.502 | 15.875 | ＜0.001 |
