# Supplementary material for: The effectiveness of the combined problem-based learning (PBL) and case-based learning (CBL) teaching method in the clinical practical teaching of thyroid disease
Source: BMC Med Educ. 2020 Oct 22;20:381. doi: 10.1186/s12909-020-02306-y (PMC7583209; doi:10.1186/s12909-020-02306-y)
Supplement: Supplementary file 6 — Additional file 6. [file 12909_2020_2306_MOESM6_ESM.docx]

**PBL联合CBL教学法在甲状腺外科临床教学中的应用课后问卷**

**The After-class Teaching Questionnaire of the Combined Problem-Based Learning (PBL) and Case-Based Learning (CBL) Teaching Method in the Clinical Practical Teaching of Thyroid Disease**

同学：您好！

本问卷是一份学术性研究问卷，是对PBL联合CBL教学法与传统授课教学法在甲状腺外科临床教学中应用效果的主管评价的情况调查，希望您能真实客观地填写问卷。问卷每个问题，根据课后改善程度，将效果分为5个等级，1=差；2=较差；3=中；4=良好；5=极好。其中对于同学们在本次课程所消耗的课余时间的问题，1代表最少的时间消耗，而5表示最高的时间消耗。

提示：本问卷所有题项均为单项选择题，选择时只需在您认同的数字窗口打“√”。

Classmate: Hello!

This questionnaire is an academic research questionnaire, which is an investigation of the supervisor's evaluation of the application effect of PBL combined with CBL teaching method and traditional teaching method in the clinical teaching of thyroid surgery. I hope you can fill in the questionnaire honestly and objectively. According to the improvement degree after class, the effect of each question in the questionnaire is divided into 5 levels, 1= worse; 2 = poor; 3 =average; 4 = good; 5 = excellent. For the question of students' spare time consumption in this course, 1 represents the lowest time consumption, while 5 represents the highest time consumption.

Tip: All questions in this questionnaire are single choice questions. You only need to tick "√" in the number window you agree with.

**一、个人基本信息：**

学生编号：

性别： 年龄：

请选择学生类型：本科同学（） 住院实习医师（）

**1. Basic Personal Information:**

Student No. :

Gender: Age:

Please select Student Type: Undergraduate student ( ) Resident Intern ( )

**二、课后教学效果调查问卷内容：**

| 描述 | 差  （少） | 较差  （较少） | 中等  (适中) | 良好  （较多） | 极好  （多） |
| --- | --- | --- | --- | --- | --- |
|  | 1 | 2 | 3 | 4 | 5 |
| 1、对学习主动性的提升情况 |  |  |  |  |  |
| 2、对本节课知识点的理解情况 |  |  |  |  |  |
| 3、师生之间的互动性的加强情况 |  |  |  |  |  |
| 4、你觉得本次课程对期末考试的帮助 |  |  |  |  |  |
| 5、沟通技巧的提升情况 |  |  |  |  |  |
| 6、临床思维能力的提升情况 |  |  |  |  |  |
| 7、自学能力的提升情况 |  |  |  |  |  |
| 8、团队合作能力的提升情况 |  |  |  |  |  |
| 9、对本次课程知识的消化吸收情况 |  |  |  |  |  |
| 10、本节课程花费自己课余时间情况 |  |  |  |  |  |

**2. After-class Teaching Effect Questionnaire contents:**

| Content | worse | poor | average | good | excellent |
| --- | --- | --- | --- | --- | --- |
|  | 1 | 2 | 3 | 4 | 5 |
| 1. Promotion of learning initiative |  |  |  |  |  |
| 2. Understanding of the knowledge points of this class |  |  |  |  |  |
| 3. Enhanced interaction between teachers and students |  |  |  |  |  |
| 4. How do you think this course will help you in the final exam |  |  |  |  |  |
| 5. Improvement of communication skills |  |  |  |  |  |
| 6. Improvement of clinical thinking ability |  |  |  |  |  |
| 7. Improvement of self-learning ability |  |  |  |  |  |
| 8. Improvement of teamwork ability |  |  |  |  |  |
| 9. Memory of knowledge of this course |  |  |  |  |  |
| 10. How much spare time spent in this course |  |  |  |  |  |
